# E-cadherin interactions are required for Langerhans cell differentiation

**DOI:** 10.1002/eji.201242654

**Published:** 2012-12-11

**Authors:** Nobuko Mayumi, Eri Watanabe, Yoshihiko Norose, Eiji Watari, Seiji Kawana, Teunis B H Geijtenbeek, Hidemi Takahashi

**Affiliations:** 1 Department of Microbiology and Immunology, Nippon Medical SchoolTokyo, Japan; 2 Department of Dermatology, Nippon Medical SchoolTokyo, Japan; 3 Department of Experimental Immunology, Academic Medical Center, University of AmsterdamAmsterdam, The Netherlands

**Keywords:** DC, DC-SIGN, E-cadherin, Langerhans cells, Langerin

## Abstract

Human skin contains the following two distinct DC subsets: (i) Langerhans cells (LCs), expressing Langerin but not DC-specific intercellular adhesion molecule-3-grabbing nonintegrin (DC-SIGN), are predominantly localized in the epidermis; and (ii) dermal DCs, expressing DC-SIGN but not Langerin, are observed mainly in the dermis. It is not known whether localization in the epidermis provides cues for LC differentiation. Here, we show that E-cadherin expressed by epidermal keratinocytes (KCs) is crucial for differentiation of LCs. Monocytes differentiated into LC-like cells in presence of IL-4, GM-CSF, and TGF-β1. However, these LC-like cells expressed not only Langerin but also DC-SIGN. Notably, co-culturing of these LC-like cells with KCs expressing E-cadherin or recombinant E-cadherin strongly decreased expression of DC-SIGN and further induced a phenotype similar to purified epidermal LCs. Moreover, pretreatment of LC-like cells with anti-E-cadherin-specific antibody completely abolished their Langerin expression, indicating the requirement of E-cadherin–E-cadherin interactions for the differentiation into Langerin^+^ cells. These findings suggest that E-cadherin expressed by KCs provide environmental cues that induce differentiation of LCs in the epidermis.

## Introduction

Skin and mucosal tissues contain two distinct DC subsets localized in distinct compartments. Langerhans cells (LCs) are localized in the mucosal epithelial layer and epidermal layer in skin. These LCs are the first DC subset to encounter pathogens upon infection. In contrast, the mucosal subepithelial layer as well as the dermis of the skin does not contain LCs but so-called subepithelial or dermal DCs 1. It is becoming clear that both DC subsets have specific functions 2.

Both subsets can be distinguished by the differential expression of pattern recognition receptors and in particular C-type lectins. LCs predominantly express a unique C-type lectin called Langerin that efficiently captures intruding pathogens such as HIV-1 and fungi 3,4, whereas DCs express the C-type lectin DC-SIGN (DC-specific intracellular adhesion molecule-3-grabbing nonintegrin) that also interacts with pathogens and is involved in immune modulation 5. Furthermore, in contrast to DCs, LCs have low or no expression of TLRs that recognize bacterial components such as TLR2 and TLR4 6,7. Although these data suggest that LCs might have a more tolerogenic function toward bacterial pathogens, their function remains unclear 8.

Geissmann et al. 1998 reported previously that peripheral blood monocytes (PBMos) may differentiate into LC-like cells when cultured with TGF-β1, GM-CSF, and IL-4; however, these LC-like cells express both DC-SIGN and Langerin 10. Thus, although TGF-β1 drives the differentiation of CD14^+^ PBMos from dermal DCs to LC-like cells, they are not identical to epidermal LCs. These studies suggest that other factors are required to differentiate monocytes toward LCs. Freshly isolated LCs from the skin express both Langerin and E-cadherin, and E-cadherin is also expressed by epidermal keratinocytes (KCs) and homotypic interactions of E-cadherin are involved in clustering between KCs and LCs 11. LC migration lowers E-cadherin expression 12, which suggests that KC–LC interactions are required within the epidermis either to retain LCs or for differentiation of LCs. Indeed, it has recently shown that a KC signal can induce proliferation of epidermal resident LCs 13.

Here, we have investigated the importance of E-cadherin interactions on the differentiation of LCs from monocytes. Our data show that E-cadherin is required to induce CD1a^+^, Langerin^+^, DC-SIGN^–^ Langerhans cells (moLCs) from monocytes. DC-SIGN^+^, Langerin^+^ LC-like cells are induced from monocytes after 3 days by culturing with TGF-β1. Co-culturing with KCs expressing E-cadherin or soluble E-cadherin decreases DC-SIGN and increases Langerin expression. The phenotypic features of these moLCs closely resembled skin epidermal LCs (primary LCs), in that they expressed LC-specific markers, CD1a, Birbeck granules, and Langerin, but lacked DC-SIGN and TLR4. These data suggest that E-cadherin interactions within the epidermis/epithelial layers are required to for differentiation of LCs. Furthermore, our E-cadherin co-culture model to generate moLCs might facilitate research into LC functions.

## Results

### TGF-β1 induces LC-like cells expressing both Langerin and DC-SIGN

**Figure 1:**
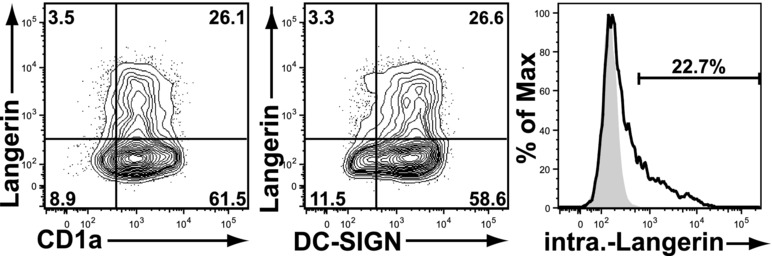
LC-like cells, generated from PBMos with GM-CSF, TGF-β1, and IL-4, express both Langerin and DC-SIGN. CD14^+^ PBMos were cultured for 6 days in the presence of 100 ng/mL GM-CSF, 10 ng/mL IL-4, and 10 ng/mL TGF-β1 as described in the *Materials and Methods*. The obtained LC-like cells were triple stained with anti-CD1a, anti-Langerin, and anti-DC-SIGN Abs and analyzed by flow cytometry. CD1a^+^ cells were gated on (left), and the surface expression of Langerin and DC-SIGN on these cells are shown (middle). CD1a-gated LC-like cells were further examined for intracellular Langerin expression (right). Results shown are representative of four independent experiments performed.

LC-like cells can be obtained from monocytes in vitro by culturing with GM-CSF, IL-4, and TGF-β1 9. However, in contrast to epidermal LCs that express Langerin but not DC-SIGN 3, the CD1a^+^ gated LC-like cells (Fig. 1A; left panel) expressed both Langerin and DC-SIGN (Fig. 1; middle panel). Also, Langerin was detected partially (22.7%) intracellularly among the LC-like cells (Fig. 1; right panel). These data suggest that monocytes can be differentiated into LC-like cells but further differentiation factors are required to be the actual LCs.

**Figure 2:**
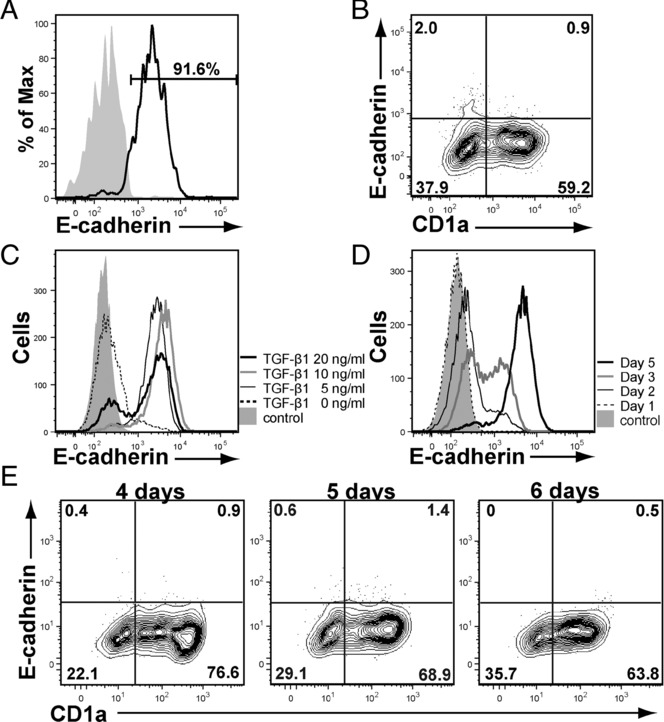
E-cadherin expresses on KCs and TGF-β1-treated LC-like cells. (A) E-cadherin expression on KCs was determined by staining with HECD-1 antibody (IgG1) and analyzed by flow cytometry. Isotype-matched antibody was used for control staining. (B) DCs generated from PBMos with 10 ng/mL IL-4, 100 ng/mL GM-CSF for 6 days (6-day-DCs) were stained with anti-CD1a and anti-E-cadherin. (C) E-cadherin-expression on PBMos cultured with IL-4, GM-CSF and various concentration of TGF-β1 (0–20 ng/mL) for 6 days is shown. (D) The kinetics of E-cadherin expressions on LC-like cells generated from PBMos with IL-4, GM-CSF, and 10 ng/mL TGF-β1 are shown. (E) Effect of addition of 10 ng/mL TGF-β1 on PBMo-derived DC precursors cultured for more than 4 days. Results shown are representative of four independent experiments.

It has been reported that freshly isolated LCs from the skin express both Langerin and E-cadherin, whereas E-cadherin is also expressed by epidermal KCs 11. Indeed, as compared with isotype-matched control staining shown as shaded histogram, E-cadherin is certainly expressed on human epidermal KCs (Fig. 2A). Next, we analyzed E-cadherin expression on monocyte-derived DCs (moDCs) induced by IL-4 and GM-CSF. These moDCs do not express any E-cadherin (Fig. 2B). Also, TGF-β1 has been shown to induce E-cadherin on moDCs 9. Therefore, we invest-igated E-cadherin expression on differentiation of monocytes to LC-like cells. E-cadherin was apparently induced on LC-like cells when monocytes were cultured with TGF-β1 from the start of culture and the peak level was observed at 10 ng/mL (Fig. 2C). Furthermore, E-cadherin expression on monocytes cultured with GM-CSF, IL-4, and 10 ng/mL TGF-β1 gradually increased after initiation of the culture, and became sufficient at around day 3 of culture and the peak level was seen at day 5 (Fig. 2D). It is important to note that after culturing monocytes with IL-4 and GM-CSF for more than 4 days E-cadherin-positive cells are no longer induced by the addition of TGF-β1 (Fig. 2E). These findings suggest that TGF-β1 stimulation is required to gain E-cadherin expression from the very early stage of DC-differentiation and cells will not achieve E-cadherin expression by TGF-β1 stimulation once they enter differentiation steps to DCs.

### E-cadherin^+^ KCs induce LC differentiation

**Figure 3:**
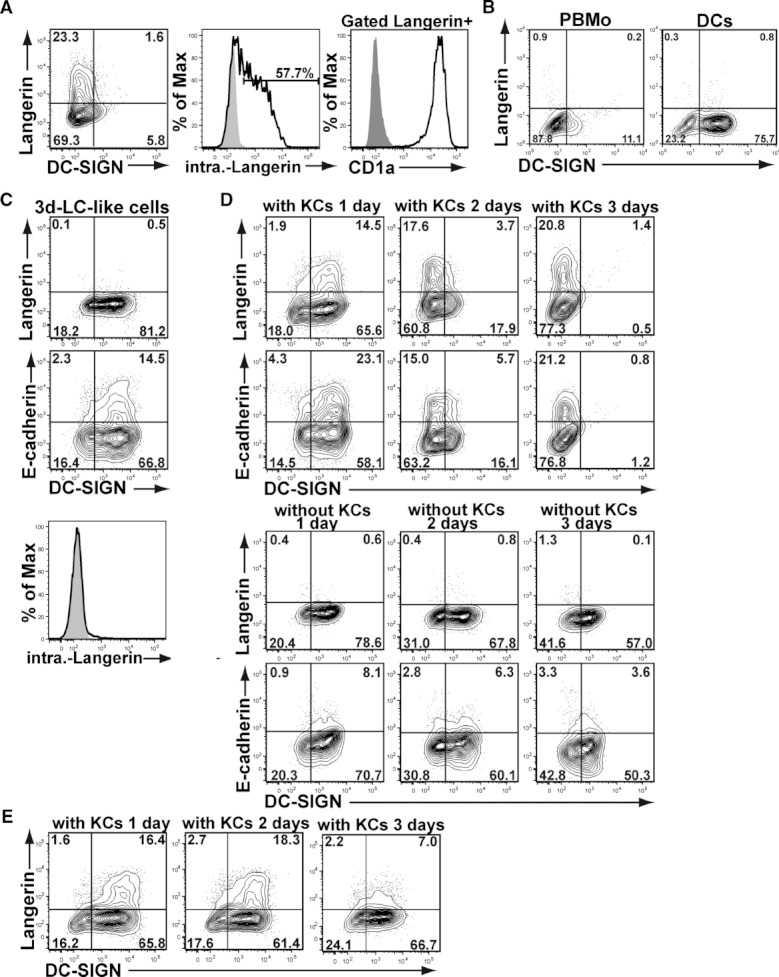
Co-culture of E-cadherin^+^ LC-like cells with E-cadherin^+^ KCs induces differentiation of LCs. (A) Three-day-cultured PBMo-derived E-cadherin^+^ LC-like cells (3-day-LC-like cells) incubated with KCs for an additional 3 days were analyzed for their Langerin and DC-SIGN expression by flow cytometry (left). The generated Langerin^+^ cells were examined their intracellular Langerin expression (middle) and stained with anti-CD1a (right). (B) PBMos co-cultured with KCs with GM-CSF, IL-4, and 10 ng/mL TGF-β1 for 6 days were stained with anti-Langerin and anti-DC-SIGN (left). Six-day-DCs generated from PBMos were co-cultured with KCs for an additional 3 days (right). (C) The expression of Langerin, DC-SIGN (top), E-cadherin (middle), and intracellular Langerin (bottom) of 3-day-LC-like cells is shown. (D) The kinetics of Langerin, DC-SIGN, and E-cadherin expressions of 3-day-LC-like cells after co-culturing with KCs was analyzed for three successive days. Top panels show alteration of Langerin and DC-SIGN expression on co-cultured 3-day-LC-like cells and upper middle panels present their E-cadherin and DC-SIGN expression. Similarly, bottom middle panels show alteration of Langerin and DC-SIGN expression on 3-day-LC-like cells without co-culturing with KCs while the bottom lanes present their E-cadherin and DC-SIGN expression. (E) E-cadherin^+^ LC-like cells culturing for 5 days were further co-cultured with KCs, and analyzed for three successive days. Results shown are representative of six independent experiments.

Next we investigated whether KCs could provide additional differentiation stimuli for generation of moLCs. DC-SIGN^+^, Langerin^+^ LC-like cells were co-cultured on monolayer of E-cadherin-expressing KCs. Notably, we could generate DC-SIGN^–^, augmented Langerin^+^ moLCs by co-culturing 3-day cultured PBMo-derived E-cadherin^+^ LC-like cells (3-day-LC-like cells) with E-cadherin^+^ KCs for an additional 2–3 days with 100 ng/mL GM-CSF (Fig. 3A; left panel). The generated Langerin^+^ cells highly expressed Langerin intracellularly (Fig. 3A; middle panel) in comparison with the LC-like cells (Fig. 1A; middle panel) and were CD1a^+^ (Fig. 3A; right panel). In contrast, we did not detect any Langerin^+^ cells by co-culturing either monocytes (PBMos) (Fig. 3B; left panel) or well-differentiated E-cadherin^–^ DCs (6-day DCs) with E-cadherin^+^ KCs (Fig. 3B; right panel) for an additional 2–3 days with 100 ng/mL GM-CSF. DC-SIGN expression on LC-like cells after 3 days of culture with GM-CSF, IL-4, and TGF-β1 (3-day-LC-like cells) (Fig. 3C) gradually decreased to background levels when co-cultured with KCs (Fig. 3D). No DC-SIGN expression is observed on moLCs after 3 days of co-culturing with KCs. Here, E-cadherin expression on those 3-day-LC-like cells interacting with KCs was slightly augmented (Fig. 3D), suggesting the augmented interactions between 3-day-LC-like cells and KCs were initiated through E-cadherin. It is of note that DC-SIGN expression gradually decreased without expressing Langerin when 3-day-LC-like cells were washed to remove TGF-β1 and further incubated with complete culture medium (CCM) plus GM-CSF (Fig. 3D). E-cadherin expression was also gradually downmodulated on those cells (Fig. 3D). The effect of KCs on LC differentiation was dependent on the differentiation stage since DC-SIGN expression on 5-day-differentiated LC-like cells expressing the highest level of E-cadherin (Fig. 2D) was not decreased when co-cultured with E-cadherin^+^ KCs (Fig. 3E). These data show that KCs are required for differentiation of LCs from monocytes and this depends on the differentiation stage, and suggest that the different compartments in skin provide different differentiation factors required for generation of moDCs or moLCs.

### E-cadherin interactions are required for LC differentiation

**Figure 4:**
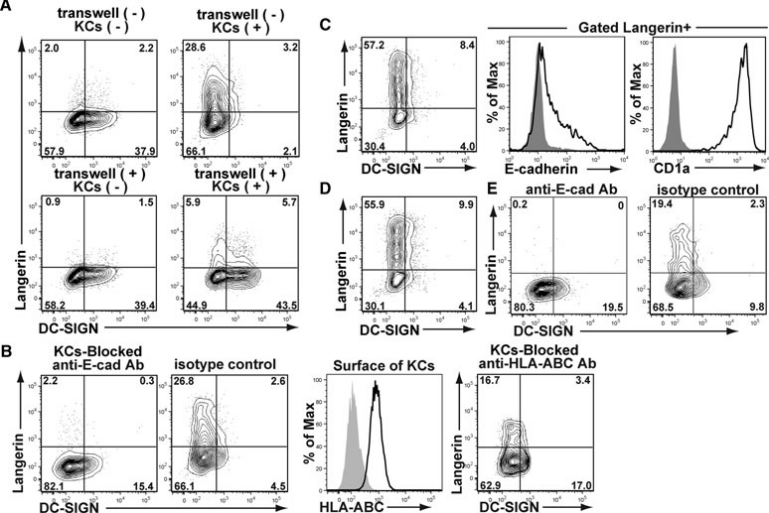
Differentiation of LC-like cells into moLCs was mediated through direct interaction with E-cadherin on KCs. (A) Three-day-LC-like cells were cultured with (right) or without (left) KCs, with (bottom) or without (top) Transwell® inserts. Cells were stained with anti-Langerin and anti-DC-SIGN. (B) Three-day-LC-like cells were cultured with KCs pretreated with anti-E-cadherin antibody or control isotype-matched antibody for an additional 3 days and stained with anti-Langerin and anti-DC-SIGN (two left-most panels). After confirming the expression of HLA-ABC on KCs, KCs were pretreated with anti-HLA-ABC antibody and co-cultured with 3-day-LC like cells (two right-most panels). (C) Three-day-LC-like cells were added to human E-cadherin-coated plates and incubated for 3 days. The generated cells were stained with anti-Langerin, anti-DC-SIGN, anti-E-cadherin, and anti-CD1a. Expression of Langerin and DC-SIGN (left), E-cadherin (middle), and CD1a (right) in Langerin^+^ cells is shown. (D) Langerin and DC-SIGN expressions of moLCs generated using murine E-cadherin-coated plates and incubated for 3 days. (E) Three-day-LC-like cells were treated with anti-E-cadherin-specific antibody for 30 min and washed three times with CCM to remove free antibody and stimulated with plate-coated purified human E-cadherin for an additional 3 days. Results shown are representative of four independent experiments.

Next we investigated whether E-cadherin on KCs is a critical factor for the induction of Langerin^+^ moLCs. We incubated 3-day-LC-like cells with E-cadherin^+^ KCs in a transwell system composed of separate wells to prevent direct interaction between the cells. LC-like cells co-cultured in the transwell system with KCs did not differentiate into moLCs since the expression of DC-SIGN was not decreased (Fig. 4A). Thus, cell–cell contact but not soluble factors is critical for LC differentiation. E-cadherin has been shown to mediate LC–KC interactions and we investigated whether E-cadherin interactions are required for LC differentiation. Pretreatment of KCs with an antihuman E-cadherin antibody blocked the induction of Langerin^+^, DC-SIGN^–^ moLCs whereas pretreatment with isotype-matched control antibody did not affect the LC differentiation (Fig. 4B; right 2 panels). Also, antihuman HLA-ABC-specific antibody, which binds to KCs, do not block the LC induction (Fig. 4B; left 2 panels). These results strongly suggest that E-cadherin interactions are required for the differentiation of LCs from monocytes.

We next investigated whether E-cadherin itself can induce LC differentiation. LC-like cells were cultured on human E-cadherin-coated plates and, notably, we could generate markedly augmented Langerin^+^, DC-SIGN^–^ moLCs by stimulation with plate-coated purified human E-cadherin (Fig. 4C). Similarly, culturing of LC-like cells with plate-coated murine E-cadherin strongly induced Langerin^+^, DC-SIGN^–^ moLCs (Fig. 4D).

Moreover, pretreatment of 3-day-LC-like cells with E-cadherin-specific antibody for 30 min totally stopped their differentiation into Langerin^+^, DC-SIGN^–^ moLCs by stimulation with plate-coated purified human E-cadherin for 3 day although pretreatment of isotype-matched antibody did not affect their differentiation (Fig. 4E). These data strongly suggest E-cadherin on KCs is required for the final differentiation steps of LCs from monocytes.

### Phenotype and characteristics of moLCs

**Figure 5:**
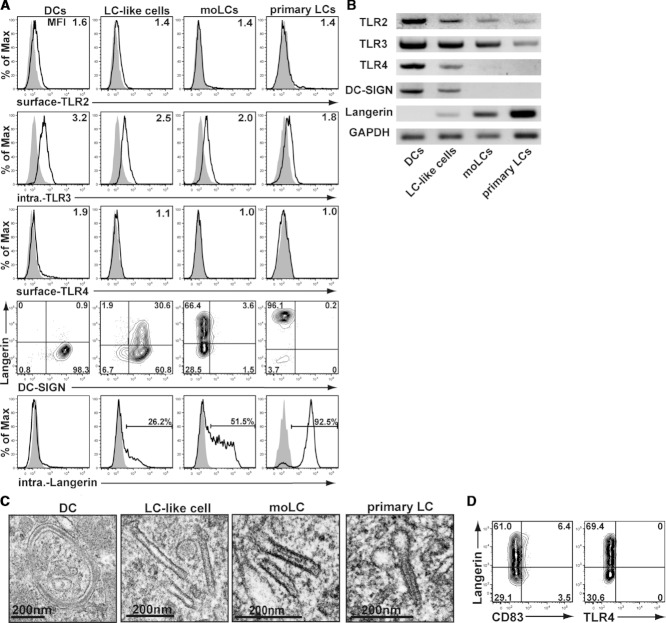
PBMo-derived moLCs have a similar phenotype to that of immature epidermal skin primary LCs. (A) PBMo-derived DCs, LC-like cells, moLCs, and skin-derived primary LCs were double-stained with anti-Langerin (both external and intracellular) and anti-TLR2 or anti-TLR3 or anti-TLR4 to analyze TLR expressions of Langerin^+^ gated cells using histograms. (B) This was confirmed by RT-PCR analysis using cell-extracted mRNA and the set of detection primers described in the *Materials and Methods*. GAPDH was used as a control. (C) Birbeck granules were identified by electron microscope analysis. (D) The obtained moLCs were double-stained with anti-Langerin, anti-CD83, and anti-TLR4 antibodies. Results shown are representative of six independent experiments.

Previously, several studies have shown that purified LCs from human skin does not express TLR4 and little or no TLR2 6,7. Here, we investigated the TLR expression of moLCs differentiated by E-cadherin in comparison with PBMo-derived DCs, LC-like cells, and skin-derived primary LCs. Similar to primary LCs, Langerin^+^ DC-SIGN^–^ moLCs did not express TLR4 and had a low expression of TLR2 and TLR3 both at the mRNA and protein level (Fig. 5A and B). Moreover, electron microscopic analysis clearly showed Birbeck granules in the PBMo-derived cultured moLCs whose precise structure is also closely resemble to the primary LCs (Fig. 5C). Furthermore, these moLCs did not express CD83, a maturation marker, and TLR4 (Fig. 5D). Thus, although the Langerin expression level was not uniform for PBMo-derived moLCs, E-cadherin-induced moLCs have a highly similar phenotype as immature epidermal skin LCs.

### Effect of stimulation with TLR agonists on moLCs

**Figure 6:**
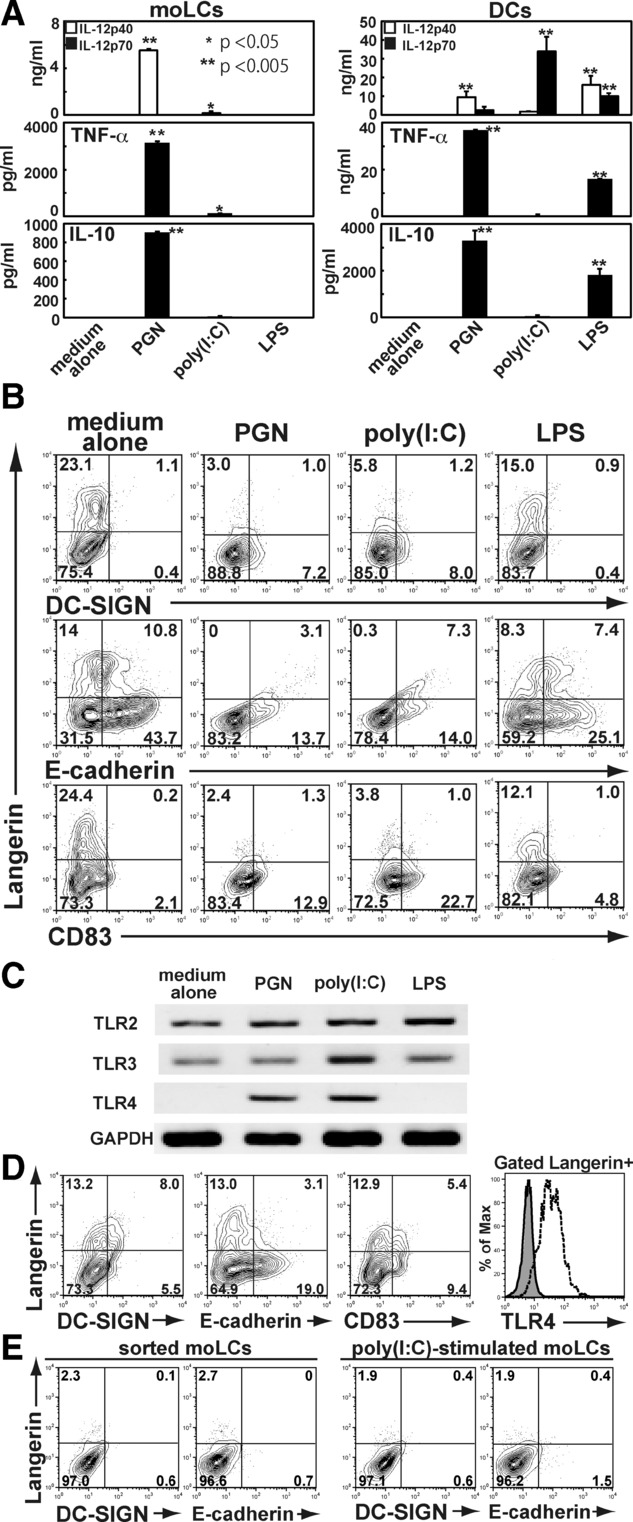
PBMo-derived, moLCs irreversibly became activated DCs via TLR–ligand stimuli. (A) PBMo-derived moLCs (left) or DCs (right) was stimulated with known TLR agonists; 20 μg/mL PGN for TLR2, 50 μg/mL poly(I:C) for TLR3, or 100 ng/mL LPS for TLR4, and cytokine production was measured by ELISA. Data are shown as the mean + SEM (*n* = 4) of results pooled from four independent experiments. *p < 0.05, ^**^p < 0.005, Student's *t*-test . (B) PBMo-derived moLCs stimulated by those TLR-ligans for 24 h were analyzed their expression of Langerin, DC-SIGN, E-cadherin, and CD83. (C) Ligand-stimulated moLCs were examined for the expression of TLRs. (D) Langerin^+^ moLCs separated by sorting with magnetic cell separator (MACS) were analyzed their expression of DC-SIGN, E-cadherin, CD83, and TLR4. (E) Sorted moLCs (left) or poly(I:C)-stimulated moLCs (right) were stimulated on a human E-cadherin-coated plate and examined their expression of Langerin and E-cadherin. Results shown are representative of four independent experiments.

It is well known that the cytokine secretion profiles in response to various stimuli are different between LC-like cells and DCs 14. We therefore stimulated E-cadherin-induced moLCs and moDCs obtained from the same monocytes by the known TLR agonists; peptidoglycan (PGN) for TLR2, poly(I:C) for TLR3, or LPS for TLR4, and cytokine production for each stimulus was measured by ELISA. Although moDCs secreted several inflammatory cytokines, such as IL-12p40, TNF-α, and IL-10 by PGN and LPS and IL-12p70 production by poly(I:C), moLCs released a very small amount of IL-12p40, TNF-α and IL-10 in response to PGN and poly(I:C), but did not respond to LPS (Fig. 6A). It should be noted that moLCs did not produce IL-12p70 by poly(I:C) stimulation.

Next we investigated expression of Langerin, DC-SIGN, E-cadherin, CD83, and TLR4 in E-cadherin-induced moLCs treated with TLR ligands. The expression of Langerin and E-cadherin was downmodulated on activated moLCs, particularly those treated with PGN and poly(I:C), but not with LPS, while the expression of CD83 and DC-SIGN appeared by ligand stimulation (Fig. 6B). These results support the TLR expression data and show that moLCs weakly respond to TLR2, and TLR3 ligands and both TLR2 and TLR3 ligands induce a phenotype switch in these cells.

Importantly, TLR4 also appeared by PGN and poly(I:C) stimulation of moLCs (Fig. 6C). Moreover, when PBMo-derived Langerin^+^ moLCs were sorted by FACSAria-II, Langerin as well as E-cadherin expression disappeared; nevertheless, DC-SIGN as well as TLR4 appeared with upregulated CD83 on those sorted moLCs (Fig. 6D). Furthermore, sorted moLCs or poly(I:C)-stimulated moLCs would not become a Langerin^+^ cells even when co-cultured on a human E-cadherin-coated plate (Fig. 6E). These findings suggest that epidermal LCs may irreversibly change into dermal DCs by pathogen intrusion through TLR signaling or by mechanical isolation, and once LCs lose their E-cadherin, they cannot recover their original epidermal LCs.

### Effect of crosslinking of E-cadherin on moLCs

**Figure 7:**
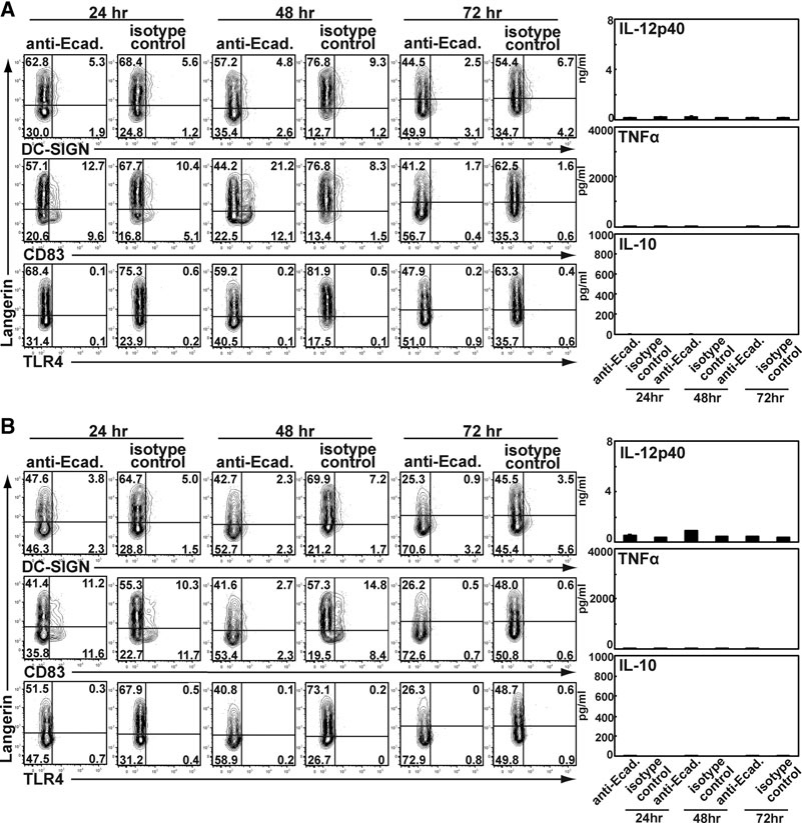
Effect of cross-linking E-cadherin on moLCs on moLC activation. (A) Purified moLCs were incubated with anti-E-cadherin-specific antibody for 30 min and plated for additional 24–72 h after extensive washing with the medium. (B) Purified moLCs were incubated on the anti-E-cadherin Ab-coated plate for additional 24–72 h after extensive washing with the medium. The expression of DC-SIGN, TLR4, and CD83 on Langerin-positive cells (left) as well as cytokine production of the culture supernatant measured by ELISA (right) are shown. Results shown are representative of six independent experiments.

Finally, we investigated the effect of crosslinking of E-cadherin on the activation of moLCs. Purified moLCs were pretreated with anti-E-cadherin-specific antibody for 30 min and plated for additional 24–72 h (Fig. 7A), or incubated directly on the anti-E-cadherin Ab-coated plate for additional 24–72 h to see the effect of crosslinking (Fig, 7B). Next we analyzed the expression of DC-SIGN, TLR4, and CD83 on Langerin positive cells. Although we did observe a slight downmodulation of Langerin and CD83 expression, both TLR4 expression and cytokine expression was not affected by crosslinking or stimulation of E-cadherin on moLCs (Fig. 7A and B). Taken together, these findings suggest that moLCs can efficiently be activated to secrete cytokines and express TLR4 via TLR signaling but not through E-cadherin crosslinking.

## Discussion

Here, we have shown that E-cadherin on KCs is required for differentiation of monocytes into LCs. Although TGF-β1-induced LC-like cells, E-cadherin triggering induced final differentiation into LCs with a phenotype similar to primary isolated LCs. These moLCs markedly resembled skin epidermal LCs, in that they expressed LC-specific markers CD1a, Birbeck granules, and Langerin but lacked DC-SIGN and TLR4. Our study strongly suggests that homotypic interactions between E-cadherin on KCs and on LCs are required for final differentiation of LCs and that the epidermis provides environmental cues for final differentiation of LCs.

Recent studies have suggested that E-cadherin can regulate LC maturation and migration 15. However, our data suggest that E-cadherin interactions are also required for differentiation into LCs. TGF-β1 is known to induce LC differentiation from monocytes and CD34^+^ cord blood cell 9,16,17. Indeed, TGF-β1-induced differentiation of monocytes into LC-like cells that expressed LC-specific markers langerin and CD1a. However, these LC-like cells also expressed DC-SIGN, which is not expressed by LCs in human tissues. Notably, co-culture of these LC-like cells on E-cadherin expressing KCs or recombinant E-cadherin-coated plate induced further differentiation and led to loss of DC-SIGN expression and increased Langerin expression. These data strongly suggest that E-cadherin on KCs is required for differentiation of LCs. Several E-cadherin ligands have been identified such as CD103 and the inhibitory killer cell lectin-like receptor G1 (KLRG) 15. Our data show that homotypic interactions are required since preincubation of LC-like cells with blocking antibodies against E-cadherin totally abolished LC differentiation during co-culture on KCs, which is compatible to the previous report showing that homotypic LC clustering can be inhibited by the addition of anti-E-cadherin mAb 18. Thus, E-cadherin interactions are not only involved in providing a niche for LCs but also for inducing final differentiation of monocytes into LCs when they migrate into the epidermis.

E-cadherin signaling can occur through Wnt/β-catenin signaling pathway since E-cadherin interacts with β-catenin and thereby limits the cytoplasmic pool of β-catenin 19. Triggering of E-cadherin might lead to an increase in cytoplasmic β-catenin and this will result in activation of Wnt signaling. Further studies are required to investigate whether Wnt signaling is involved in LC differentiation. Interestingly, Wnt signaling is important in developmental processes and might therefore also play a role in LC differentiation.

E-cadherin-induced moLCs had a similar expression profile of TLRs as isolated epidermal LCs 6. Epidermal LCs do not express TLR4 and low levels of TLR2, which prevents continuous activation of LCs to commensal microbes. Our data show that E-cadherin^+^ moLCs do not express TLR4, further supporting their LC phenotype.

E-cadherin expression of moLCs decreased after maturation by TLR ligands PGN or poly(I:C) but not with LPS. Such reduction of E-cadherin expression on moLCs may decrease their ability to interact with epidermal KCs and release them for migration into the dermis. These findings suggest that LCs seem to be tethered by KCs through E-cadherin chains in the epidermis. Indeed, Choroo et al. 13 have recently reported that LCs reside within the epidermal region in a steady state with the assistance of a KC signal that might be mediated through E-cadherin–E-cadherin chain-interaction between LCs and KCs.

Although skin-derived LCs can be obtained directly by the treatment of various enzymes, such as collagenase and trypsin, these vigorous steps can affect epidermal LCs and low yields are observed. We have established a method to generate immature Langerin^+^, DC_-_SIGN^–^, CD83^–^ cells from monocytes that have similar phenotype as primary LCs. The current procedure shown here may offer naïve, unstimulated LCs that will provide an excellent tool to analyze the actual features of LCs. Our data have identified a novel role for E-cadherin in differentiation of LCs. Inflammation will attract monocytes into epidermis and E-cadherin on KCs as well as TGF-β1 will provide the necessary signals to differentiate monocytes into LCs. This study provides further information into the molecular mechanisms that govern LC differentiation and the function of E-cadherin homotypic interactions.

## Materials and methods

### Reagents

The medium used for culturing cells was RPMI1640-based CCM 20 supplemented with 50 mM 2-ME (Sigma-Aldrich, St. Louis, MO, USA), 2 mM L-glutamine (Gibco BRL, Grand Island, NY, USA), 100 U/mL penicillin (Invitrogen Life Technologies, Carlsbad, CA, USA), 100 μg/mL streptomycin (Invitrogen), and 10% heat-inactivated FCS (Hyclone, Logan, UT, USA). Recombinant human GM-CSF was purchased from PeproTech (Rocky Hill, NJ, USA), recombinant human IL-4 from Biosource International (Camarillo, CA, USA), and recombinant human TGF-β1 from R&D Systems (Minneapolis, MN, USA). As specific stimuli for TLRs, LPS (026:B6) was obtained from Sigma-Aldrich, PGN was purchased from Fluka (Buchs SG, Switzerland), and poly(I:C) from Amersham Biosciences (Piscataway, NJ, USA). APC-conjugated anti-CD1a mAb (HI149) was purchased from BD PharMingen (San Diego, CA, USA), and FITC-conjugated anti-CD83 (HB15e) was from Biolegend (San Diego, CA, USA), anti-E-cadherin (HECD-1; IgG1) was from Takara-bio (Shiga, Japan), FITC-conjugated anti-DC-SIGN was from R&D Systems, and anti-Langerin (DCGM4) as well as PE-conjugated anti-Langerin (DCGM4) and anti-HLA-ABC (B9.12.1) were from Immunotech (Marseille, France). Biotin-conjugated anti-TLR2 (TL2.1), purified anti-TLR3 (TLR3.7), and Alexa Fluor®488-conjugated anti-TLR4 (HTA125) were purchased from eBioscience (San Diego, CA, USA). FITC-conjugated goat antimouse IgG antibody and PE-conjugated goat antimouse IgG antibody were obtained from Beckman Coulter (Fullerton, CA, USA).

### PBMos isolation and culture, epidermis primary LCs isolation, and human epidermal KCs culture

LC-like cells from PBMos were obtained as described previously 6 with the following modifications. Briefly, to obtain, CD14^+^ PBMos were isolated from peripheral blood of healthy volunteers using a human monocytes enrichment kit (StemCell Technologies, Vancouver, CA, USA). To induce moDCs, CD14^+^ PBMos were cultured in CCM supplemented with 100 ng/mL GM-CSF and 10 ng/mL IL-4, and, to obtain LC-like cells, CD14^+^ PBMos were cultured in CCM supplemented on day 0 with 100 ng/mL GM-CSF, 10 ng/mL IL-4, and 10 ng/mL TGF-β1.

Primary LCs were isolated based on the following procedure 3, with slight modifications. Normal healthy adult skin obtained from plastic surgery was used within 3 h after the operation. Three-millimeter-thick slices of skin, containing the epidermis and dermis, were obtained by using a dermatome. The slices were incubated with Dispase II (1 mg/mL, Roche Diagnostics, Branford, CT, USA) in IMDM (Invitrogen), 10% FCS, and gentamycine (10 mg/mL) for 2 h at 37°C. Epidermis was mechanically separated, washed it in medium and cut it into 1 mm^2^ pieces. Emigrant LCs were generated by floating the epidermis on IMDM, 10% FCS, 10 mg/mL gentamycin, and 800 U/mL GM-CSF (PeproTech). After 3 days, the migrated cells were layered on a Ficoll gradient and cultured them at 0.5 × 106 /mL in IMDM, 10% FCS, 10 mg/mL gentamycine, and 800 U/mL GM-CSF. Immature primary LCs were isolated by incubating epidermal sheets in PBS containing DNaseI (20 U/mL; Roche Applied Science, Indianapolis, IN, USA) and either trypsin (0.05% Beckton Dickinson, Franklin Lakes, NJ, USA) or collagenase blend F (0.25%, Sigma-Aldrich) for 30 min at 20–22°C. FCS was used to inactivate trypsin digestion and generated a single-cell suspension. Then LCs were selected from layered cells on a Ficoll gradient using CD1a-labeled immunomagnetic microbeads (Miltenyi Biotec, Aubum, CA, USA).

Human epidermal KCs (Kurabo, Osaka, Japan) were maintained in KC culture medium (EpiLfe-KG2; Kurabo), and then cultured KCs were plated in 24-well culture plates and further cultured until confluence. PBMo-derived LC-like cells were then loaded onto KC culture plates at a concentration of 1 × 10^5^ cell/mL in CCM supplemented with 100 ng/mL GM-CSF, with or without polycarbonate membrane inserts (Corning, NY, USA).

To perform RT-PCR or electron microscopic analysis, the obtained cells were stained with anti-Langerin and Langerin^+^ cells were purified using Rat antimouse IgG microbeads (Miltenyi Biotec.) and a magnetic cell separator (MACS; Miltenyi Biotec.). In some experiments, 3-day-cultured LC-like cells were plated on human E-cad-Fc 21 coated 24-well plate kindly provided by Celagix, Res. Ltd (Kanagawa, Japan) at a concentration of 1 × 10^5^ cell/mL in CCM supplemented with 100 ng/mL GM-CSF and further cultured for an additional 3 days to obtain Langerin^+^ DC-SIGN^–^ moLCs. This study was approved by the Review Board of Nippon Medical School and that all human participants gave written informed consent.

### Flow cytometry analysis

Cells were stained with the relevant antibody on ice for 30 min in PBS with 2% FCS and 0.01 M sodium azide (PBS-based medium), washed twice, and resuspended in PBS-based medium. For secondary staining, after washing twice, cells were incubated with an appropriate secondary antibody for 30 min and resuspended in PBS-based medium. For intracellular staining of Langerin and TLR3, cells were fixed and permeabilized with Cytofix/Cytoperm solution (BD Biosciences, Mountain View, CA, USA) for 20 min on ice. After washing twice with Perm/Wash solution (BD Biosciences), cells were incubated with AB-type serum to prevent nonspecific binding for 30 min and further incubated with FITC-conjugated anti-Langerin or anti-TLR3 for 30 min on ice in the dark. Cells stained by anti-Langerin were resuspended in PBS-based medium. For secondary staining of TLR3, after washing twice, cells were incubated with PE-conjugated secondary antimouse IgG for 30 min on ice in the dark and resuspended in PBS-based medium. Stained cells were then analyzed with FACSCantoII (BD Biosciences) using FlowJo software (TreeStar, Ashland, OR, USA). Live cells were gated based on propidium iodide gating, except for intracellular staining of cells.

### Blocking of E-cadherin molecules

To block direct cell-to-cell interaction of 3-day-cultured LC-like cells (3-day-LC-like cells) with KCs, full-seated KCs on a 24-well culture plate were pretreated with 200 μL of 0.2 mg/mL anti-E-cadherin mAb (HECD-1; IgG1) at 37°C for 1 h and then 3-day-LC-like cells were loaded for further incubation. Isotype-matched control IgG1 antibody (clone 11711) was purchased from R&D Systems.

### Electron microscopy

Cells were fixed in 2.5% glutaraldehyde/0.1 M PB (pH 7.3), 1% osmium tetroxide/0.1 M PB. The sample was then dehydrated with a series of graded ethanol and polymerized with Epoxn-812 resin (TAAB Lab., Berks, UK). Thin sections were made using an ultramicrobome (Leica, Solms, Germany), stained with uranyl acetate and lead citrate, and observed with an electron microscope (JEOL-1010; Nihon-Denshi, Tokyo, Japan).RT-PCR.

### Reverse transcription-polymerase chain reaction (RT-PCR)

Total RNA was extracted from 3 × 10^5^ cells of each cell preparation using RNeasy Kit (Qiagen, Hilden, Germany), and first-strand DNA was synthesized as described previously 22. Transcripts of TLRs as well as the housekeeping gene, GAPDH, were amplified by PCR reaction. The primer sets were as follows: GAPDH sense, 5′-GCC TCA AGA TCA TCA GCA ATG C-3′; GAPDH antisense, 5′-ATG CCA GTG AGC TTC CCG TTC-3′; TLR2 sense, 5′-CCC TGG GCA GTC TTG AAC ATT-3′; TLR2 antisense, 5′-GCC TCC GGA TTG TTA ACG TTT-3′; TLR3 sense, 5′-AGG ATT GGG TCT GGG AAC AT-3′; TLR3 antisense, 5′-CTG GAA TCT CCT CAA GGA AAA C-3′; TLR4 sense, 5′-TGG TGT CCC AGC ACT TCA TC-3′; TLR4 antisense, 5′-CTG CAT ATC TAG TGC ACC ATG G-3′; DC-SIGN sense, 5′-GAG CTT AGC AGG GTG TCT TG-3; DC-SIGN antisense, 5′-GCA GGC GGT GAT GGA GTC GT-3; Langerin sense, 5′-CGC ACT TCA CTG TGG ACA AA-3; Langerin antisense, 5′-GAA TCC AGG GTG CTG ATG TT-3. After 35 cycles of PCR, the PCR products were resolved by electrophoresis in agarose gels and visualized by ethidium bromide staining using a UV light source.

### Measurement of cytokine production by ELISA

Cytokine production in the supernatant was measured by ELISA kit for human IL-12p70, TNF-α, and IL-10 (R&D Systems), as well as for human IL-12p40 (Biolegend).

### Statistical analysis

The results were analyzed using Student's *t*-test and the results are presented as the mean ± SEM. Differences at *p* < 0.05 were considered significant.

## Abbreviations

CCM: complete culture medium
KC: keratinocyte
moDC: monocyte-derived DC
moLC: monocyte-derived LC
PBMo: peripheral blood monocyte
